# Impact of an Oral Health Education Program on the Oral Health Literacy of Refugees

**DOI:** 10.1007/s10903-024-01594-6

**Published:** 2024-04-08

**Authors:** Romana Muller, Lisa Bilich, Merri Jones

**Affiliations:** 1 Missouri School of Dentistry & Oral Health, St. Louis Dental Center, A.T. Still University1500 Park Ave, St. Louis, MO 63104 USA; 2 Eastern Washington University, 310 N. Riverpoint Blvd. Box E, Spokane, WA 99202 USA

**Keywords:** Oral health education, Oral health literacy, Refugees and immigrants, Oral health literacy assessment, Communication barriers, Limited English proficiency

## Abstract

Inadequate comprehension of healthcare information contributes to poor health outcomes. Ethnic minorities are one of the populations most affected by low health and oral health literacy (OHL). The hypothesis of the current study was that an oral health education program (OHEP) can improve the OHL, oral health awareness and behaviors of refugees. We also hypothesized that there will be a difference between OHL in English and native language in the Pre-intervention phase. Fifty-two adult refugees participated in an educational program that included a comprehensive and culturally sensitive PowerPoint presentation and hands-on learning activities on oral health topics. The study used a cross-sectional pre-post study design. Before the program (Pre-intervention group), participants completed 2 surveys: Sociodemographic Survey and Oral Health Perceptions of Refugees in a bilingual format, and the Estimate of Oral Health Literacy-Bilingual40 (EOHL-BL40) in English, and in the participants’ native language. Immediately after an OHEP (Post-intervention group), participants completed the EOHL-BL40 survey in English only. Two weeks after OHEP (Follow-up intervention), participants completed again the Sociodemographic Survey and Oral Health Perceptions of Refugees and the Estimate of Oral Health Literacy-Bilingual40 (EOHL-BL40) in English only. The mean percentage of words understood on Pre-intervention EOHL-BL40 survey were significantly higher in native languages (47.3% ±3.7%) compared with survey in English (15.3% ±1.2%, *P* < 0.001). Post-intervention scores, immediately after educational program, were higher (28.6% ±2.4%), *P* < 0.001) for survey in English compared with the Pre-intervention (15.3% ±1.2%). The follow-up (2 weeks later) scores (25.5% ±2.4) were slightly lower than Post-intervention but still significantly higher (*P* < 0.001) than Pre-intervention. Results for both Sociodemographic Survey and Oral Health Perceptions of Refugees surveys were similar: the OHEP positively affected oral health awareness and oral health behaviors. Results of the study suggested that OHEP may improve oral health literacy, oral health awareness, and personal oral hygiene practices of refugees.

**Supplementary Information:**

The online version contains supplementary material available at 10.1007/s10903-024-01594-6.

## Background

Poor oral health is considered among the main health issues for immigrants and refugees, as access to dental health care services is often compromised by multiple factors that include language barriers and low oral health literacy (OHL) [1]. Only 1 in 10 adults in the United States fully comprehend health-related information [2] and, thus, are at risk of not accessing or benefiting from the healthcare system [3]. This phenomenon was first mentioned in a report from The Agency for Healthcare Research and Quality. The study identified that that 9 in 10 adults in the US had below basic health literacy (HL) skills. The study included immigrants [4, 5].

Health literacy is defined as the “degree to which individuals have the ability to find, understand, and use information and services to inform health-related decisions and actions for themselves and others” [6]. Inadequate HL can have negative effects on the overall health of individuals, health outcomes, healthcare access, and patient-provider communication [7, 8]. Moreover, low HL has contributed to an annual increase in healthcare costs by $100-$238 billion [9]. There are numerous studies that investigated how low HL increases healthcare costs. Herndon and colleagues (2011) reviewed research that investigated the link between utilization of the Emergency Department (ED) and the level of patients’ HL. The authors found a connection between low HL of participants where low HL contributed to higher utilization of ED and incurrence of higher ED cost [10]. Oral health literacy is closely related to HL and is defined as the “degree to which individuals have the capacity to obtain, process, and understand basic oral health information and services needed to make appropriate health decisions” [8].

According to data from the 2019 American Community Survey, nearly 1 in 5 individuals residing in the United States spoke a language other than English at home [11]. In addition, 350 different languages are spoken by ethnically diverse individuals [12]. Thus, ethnic minorities are one of the populations most affected by low HL and OHL [13]. Despite this variety of nonnative English speakers, OHL assessment studies have historically focused on English and Spanish speaking populations [14–16], and other languages have typically been excluded [16]. In a 2012 study, it had been shown that communication with non-English speaking patients was challenging for students working in dental school clinics [17]. In another study, patients with limited English proficiency were more likely to have poorer knowledge of dental terms than English speakers [18].

Various tools have been developed and validated for assessment of HL and OHL. There are over twenty OHL assessment tools used in dentistry, varied in what they are designed to measure. For example, the REALD-30 and REALMD-20 only measures reading literacy and pronunciation, while the HeLD and OHLA-B measure reading literacy, pronunciation, comprehension and numeracy [19]. Many of the current OHL tools were modeled after ones designed for use in medicine. For example, the Rapid Estimate of Adult Literacy in Medicine, which is word recognition and reading test, and the Test of Functional Health Literacy in Adults, which comprehensively tests a patient’s ability to read prescriptions, were developed to assess health literacy in medical patients [20]. The OHL counterparts of these tools, the Rapid Estimate of Adult Literacy in Dentistry-30 (REALD-30) word recognition test and the Test of Functional Health Literacy in Dentistry, have been used in numerous studies [20–23]. However, some argue that tools such as the REALD-30 only evaluate the patients’ reading skills and may not indicate their true OHL level [22–24]. In a 2014 study, Khan et al. [23] compared a REALD-30-word recognition with a REALD-30 comprehension assessment and reported a significant difference in outcomes from the word recognition test alone. This finding suggests that testing understanding of dental terms based purely on word recognition may not be the most accurate measurement of OHL.

The development and delivery of an oral health education program (OHEP) may be another way to improve OHL in non-native English speakers. However, to our knowledge, such programs designed to specifically increase OHL and oral health awareness for foreign-born populations are rare, and few studies have investigated patient perceptions of oral health after they have completed an OHEP [25].

The purpose of the current study was to investigate the effect of an OHEP on the OHL of refugees. In addition to using a modified version of the REALD-30 survey to assess OHL, we also used data from sociodemographic information and from self-reported oral health awareness and oral health behavior such as personal oral hygiene practices, to evaluate the effect of the OHEP on oral health practices, perception of oral health, and values placed on disease prevention. The terms *“oral health awareness”* and *“oral health behaviors*” are routinely used in dentistry. For example, the American Dental Association in commemoration of the Federation Dentaire Internationale’s Word Oral Health Day stated that *oral health awareness* promotes good oral hygiene practices for adults and children and illustrates the importance of oral health in maintaining general health and well-being [26]. The Encyclopedia of Public Health describes *oral health behavior* as a complex effect on individual oral health of oral hygiene habits, nutritional preferences, and the pattern of a person’s utilization of dental services [27].

We hypothesized that the OHEP will improve the OHL, oral health awareness and behaviors of refugees. We also hypothesized that there will be a difference between OHL in English and native language in the Pre-intervention phase. We conducted our investigation to answer the following research questions: (1) What is the effect of an OHEP on OHL? (2) Is there a difference between OHL when tested in English and in native language? (3) What is the effect of OHEP on oral health awareness? (4) What is the effect of OHEP on oral health behavior? (5) What are the demographics of the refugees related to oral health and dental care access?

## Methods

### Study Participants

The study was approved by Eastern Washington University’s Institutional Review Board (approval no. HS-4960) before participant recruitment, ensuring compliance with ethical guidelines and federal regulations. We used convenience sampling with a purposive sampling method to recruit participants. A total of (*N* = 98) participants who were able to provide consent and attest to understanding the project goals were initially recruited for this study. Participation was completely voluntary. All participants were more than 18-year-old refugees enrolled in an English as a second language (ESL) course at the International Institute of St. Louis (IISTL), Missouri. Furthermore, participants had to have an intermediate level of English proficiency, confirmed by ESL teachers, and be able to complete all stages of the study. Participants who met our inclusion criteria were asked to sign an approved informed consent form before starting the study. However, those who missed any part of the study were excluded from the final analyses. Thus, the final group consisted of 52 immigrants (*N* = 52). Participants were asked to complete printed purpose-made surveys that assessed demographic and health characteristics, and understanding of oral health terminology. All surveys and written documents used in the study were translated to the native language of the participants using a translation service or qualified volunteer translators. Volunteers who helped distribute and collect surveys received training prior to the initiation of data collection.

The first part of the project was a pilot study with *N* = 42 participants from different countries who were asked to complete the Sociodemographic and Oral Health Perceptions of Refugees survey. This was a separate population from the one that participated in the current study (*N* = 52). The purpose of the pilot study was to assess its reliability in understanding the questions posed in order to give an answer. The focus of the pilot study was to identify potential unclear or confusing wording that could lead to misunderstandings, as well as to measure the time for the survey administration. The administration of the survey took less than 1 h.

### Study Surveys

Two surveys were used to evaluate the effect of the OHEP on OHL and to test our hypothesis that OHEP can improve the OHL, oral health awareness and behaviors of refugees. We also hypothesized that there will be a gap between the knowledge of dental terms when tested in English and in the native language. The first questionnaire was a Sociodemographic and Oral Health Perceptions of Refugees survey presented in Supplemental Table 1. The 30-item survey was created specifically for the current study and asked for demographic information (10 items), dental history (11 items), and self-reported oral hygiene practices and perceptions of oral health (9 items). Since it was an original survey, reliability and validity were tested in the pilot study. The Cronbach’s Alpha (α) Coefficients were used to determine the internal consistency (reliability) of the 3 categories of items—demographic, dental history, and self-reported oral hygiene practices and perceptions of oral health—and the overall internal consistency. Results for Cronbach’s α > 0.9 indicated good internal consistency. The Pearson Correlation Coefficients were used to test the survey validity between the individual survey items and the total score. Positive correlations (*r* range 0.75–0.91, all *P* < 0.01) were found between the individual items and the total score.

The second survey used in this study was the EOHL-BL40 (Table 1), which is a modified version of the REALD-30 published previously [14]. We obtained permission from the original authors to use the REALD-30 and modify it by adding 10 additional terms that are commonly used in patient-provider communication. This modification allowed broader assessment of OHL. The 40-word recognition survey was used to evaluate the effects of the OHEP on the OHL of participants and their retention of information from the educational program over time. For the purpose of the current study, we assessed participants’ comprehension of dental terms in two languages. By using a bilingual format, we could evaluate familiarity with dental terms in English and in the participant’s native language. More specifically, the EOHL-BL40 asked participants to read list of words and circle only the words that they understood and knew the meaning of either in English, in their native language, or in both languages. Prior to taking the survey, the participants were educated on what comprehension meant and given examples of the difference between word comprehension and word recognition.

**Table 1:** Estimate of oral health literacy bilingual-40 (EOHL-BL40) that was administered to participants pre-intervention in English and Native language, and at post-intervention and follow-up in English only

Instructions: Please circle only words which you know and understand. Please do not guess, if you only know the word in one language, circle only the word in which language you understand it.			
Sugar	Abscess	Cellulitis	Calculus
Smoking	Extraction	Fistula	Gingiva
Floss (noun)	Denture	Temperomandibular	Malocclusion
Brush teeth (verb)	Enamel	Tooth decay	Incipient
Pulp	Dentition	Apicoectomy	Root canal
Fluoride	Periodontal	Composite	Toothbrush
Braces	Sealant	Amalgam	Bristles
Genetics	Hypoplasia	Sulcus	Periodontal ligament
Restoration	Halitosis	Plaque (Biofilm)	Cementum
Bruxism	Analgesia	Anesthetic	Implant

Our explanation of the difference between word recognition and word comprehension could be related to Scarborough’s Reading Rope model that demonstrates the importance of both word recognition and language comprehension to fully understand words during reading. Word recognition includes sight recognition and language comprehension is connected to background and literacy knowledge. The model illustrates language comprehension and word recognition as separate ropes which, when woven together, become skilled reading [28]. We used examples of familiar words to demonstrate the difference between word recognition and word comprehension. For example, we wrote the word *sugar* on the blackboard and asked the group if they had seen this word before. They raised their hands. We explained that is word recognition. Then we asked them what the word *sugar* meant, and some responded that sugar is sweet tasting powder. We pointed out that the understanding of the meaning behind the word is comprehension and asked the participants to apply the principle to the word assessment by only circling words that they understand the meaning of. Total number of words circled in the assessment was the final score for each participant.

### Study Design

**Fig. 1:**
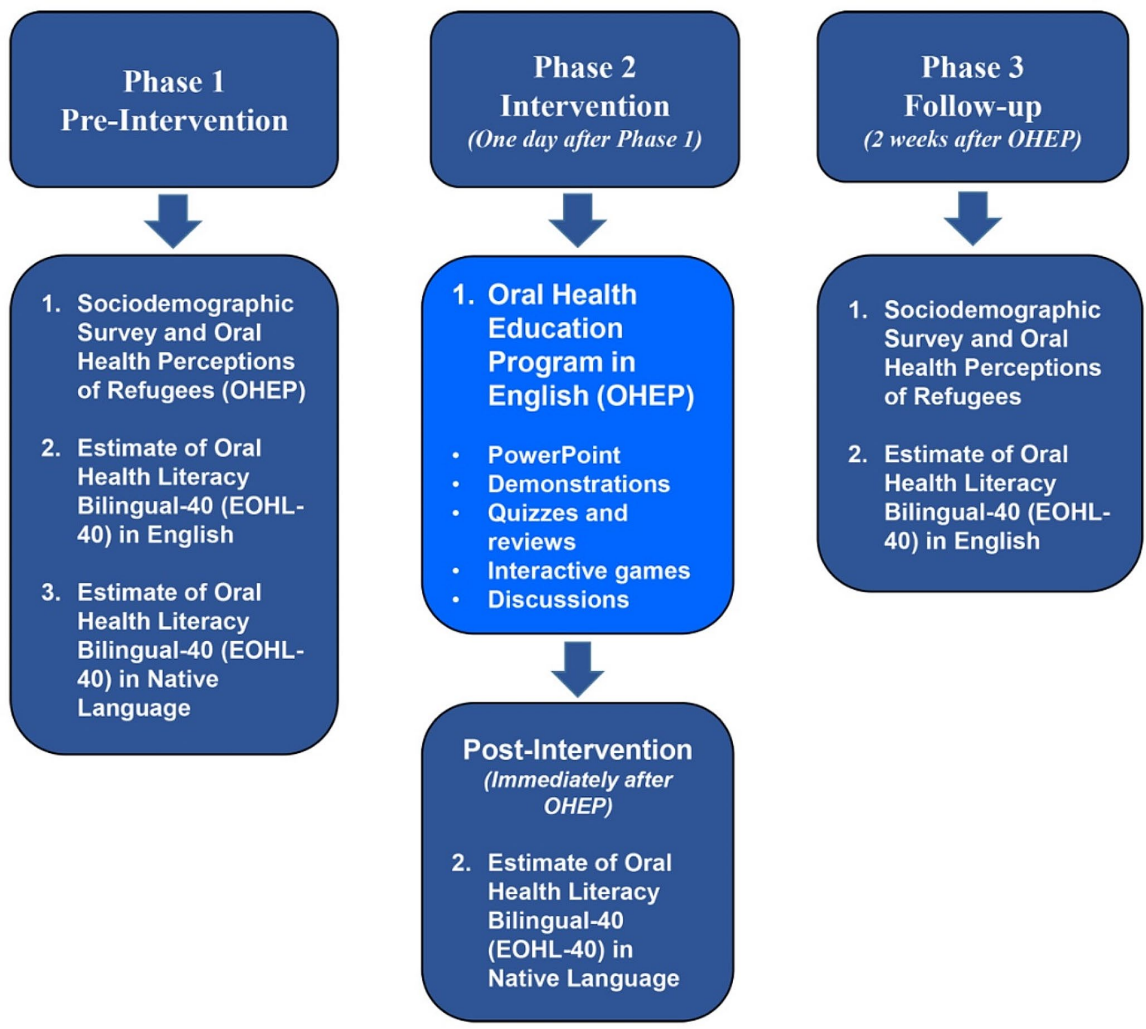
Schematic of three-phase study design

The project used a cross-sectional pre-post design to evaluate the effect of an OHEP on the OHL of immigrants (Fig. 1). Before the OHEP intervention, participants completed 2 surveys (Pre-intervention): a Sociodemographic and Oral Health Perceptions of Refugees survey (Supplemental Table 1), and the Estimate of Oral Health Literacy-Bilingual40 (EOHL-BL40) survey (Table 1). The EOHL-BL40 was administered in the participant’s native language and in English. The next day, participants completed the OHEP, which included a comprehensive PowerPoint presentation that was developed and presented by the principal investigator about dental terminology, dental procedures, and oral disease prevention in English. Participants also discussed presented topics and practiced hands-on activities. Immediately after the OHEP (Post-intervention), participants completed the EOHL-BL40 in English only to determine changes in their OHL. Two weeks following OHEP (Follow-up intervention), participants completed again the 2 Pre-intervention surveys (*oral health practices and perceptions of refugees* section of the Sociodemographic and Oral Health Perceptions of Refugees survey and EOHL-BL40 survey in English) to determine long-term retention of knowledge of dental terms and to identify changes in patient perceptions of oral health and personal oral hygiene practices over the past two weeks. The two-week follow-up period was selected due to the fact that it takes at least 18 days for an individual to adopt a habit [29]. We wanted to give the participants enough time to get accustomed to brushing and flossing before we asked questions about oral health practices again. We also took into consideration the availability of participants and facilities at the IISTL.

The EOHL-BL40 was administered in English only because the OHEP was delivered in English and not in the native languages of participants. Successful distribution of study surveys required close coordination and collaboration with the ESL program director and instructors, who also helped with organization and collection of surveys.

### Oral Health Education Program

**Fig. 2:**
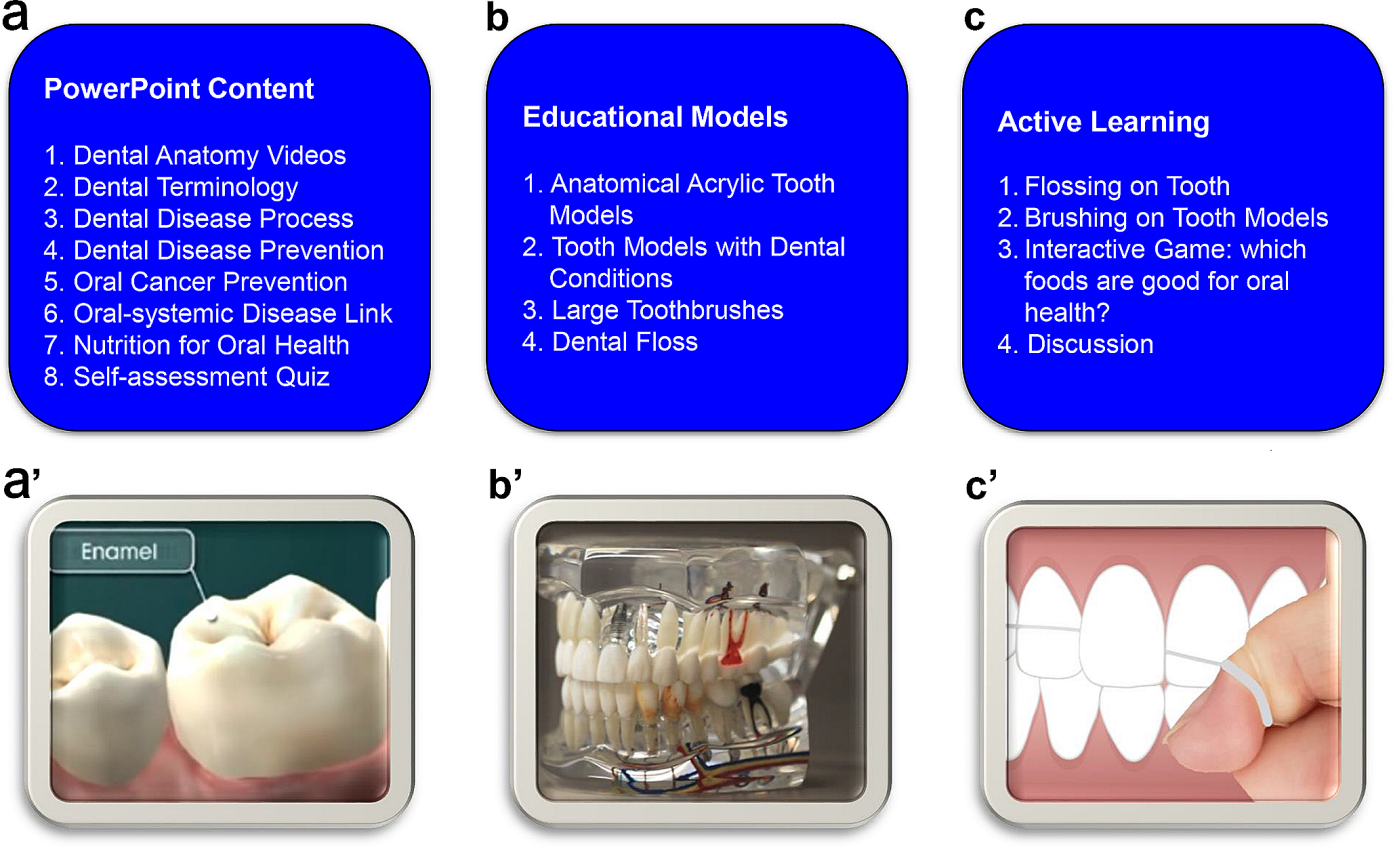
Didactic components of oral health education program for refugees (**a-c**). Panels **a**’, **b**’ and **c**’ are examples of educational content: **a**’- You Tube video on tooth anatomy. Video available at https://www.youtube.com/watch?v=rDxatqUbkVk; **b**’- Tooth model with dental conditions (image courtesy Pixabay); **c**’- Flossing technique taught during the education program (image courtesy Pixabay)

The OHEP is a new curriculum that was developed specifically for the current study. The main part of the program was PowerPoint presentation created by the researchers in English at an intermediate comprehension level or below to ensure that participants were able to understand the content (Fig. 2). The PowerPoint presentation was done in person and included visual and media information, including basic vocabulary related to oral health and dental care. The cultural competence of the presentation was verified in collaboration with the IISTL during the development phase of the OHEP. The content of the PowerPoint was reviewed for appropriate use of images by an IISTL employee with many years of experience working with refugees from various cultures. One concern was if the presentation contained outline of a human body suggesting a female form, according to our collaborator, that may not be considered an appropriate image in some cultures. The presentation included multicultural oral health topics such as dangers of tobacco use which can vary from culture to culture. For example, we presented information about the risks of chewing betel quid which is a nut combined with tobacco leaf that is a product popular in many Asian cultures. According to the World Health Organization habitual betel quid chewing can cause oral cancer [30].

The OHEP also included a visual demonstration and hands-on activities to educate participants about correct brushing and flossing techniques. Participants were given an opportunity to ask questions and practice brushing and flossing on plastic models of teeth under the supervision of registered dental hygienists. Other topics of the presentation and discussion included the basic anatomy of teeth and the oral cavity; the etiology of dental and periodontal disease; disease prevention and health promotion; the oral-systemic link; oral cancer risks; dietary effects on oral health; disease progression; restorative dentistry; and basic information about prosthodontic, endodontic, orthodontic, and periodontal dentistry. After each topic, there was a short self-assessment quiz with review questions related to the topic. We elicited participation by showing images of various foods and asking whether the food is beneficial, or not, to oral health. For every correct answer the group received a point. Participants had a chance to observe demonstration of correct brushing and flossing and try the technique themselves on plastic models. In addition, OHEP included information about cultural and generational differences in oral hygiene practices in different countries. A list of school-based dental and dental hygiene clinics providing free or discounted services was also distributed during the OHEP. The session, held in person, was completed in one day and took approximately 2 h.

### Statistical Analysis

Data were organized into spreadsheets using Microsoft Excel. Participant responses to the 2 surveys were summarized using frequency and percentage or mean, and standard deviation (SD) or standard error (SE). Paired *t* tests were used to compare participant scores on the EOHL-BL40 at the 3 study time points (Pre-intervention, Post-intervention, and Follow-up intervention) and to compare changes in oral health practices and perceptions from the sociodemographic survey and oral health perceptions of refugees. A paired *t* test was also used to compare Pre-intervention scores on the EOHL-BL40 between the participant’s native language and English. Descriptive and correlational statistical analyses were performed using SPSS statistical software version 21.0 (IBM Corp., Armonk, NY). A *P* < 0.05 was considered statistically significant. Figures were prepared using GraphPad Prism 10 (GraphPad Software, La Jolla, California), Adobe Photoshop CC 2023, and Adobe Illustrator CC 2023 (Adobe Systems, San Jose, California).

## Results

### Demographic Characteristics and Dental History of Participants

**Fig. 3:**
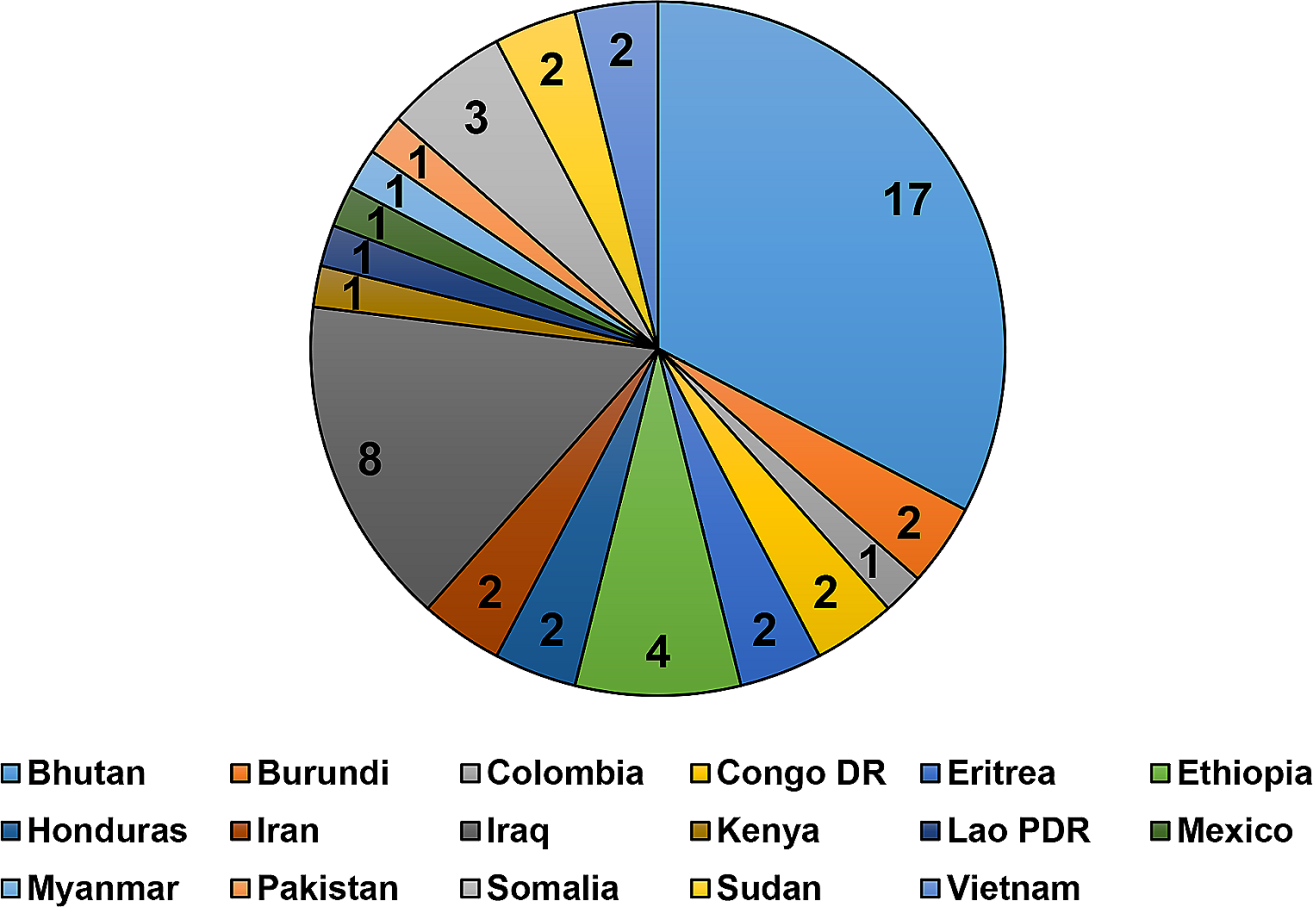
Numbers of refugees of various ethnicities (*N* = 52)

Fifty-two adult refugees and immigrants completed the study. Participants had resided in the United States from 2 months to 23 years [mean (SD) = 4.7 (5.7) years]. Seventeen different nationalities were reported (Fig. 3).

**Fig. 4:**
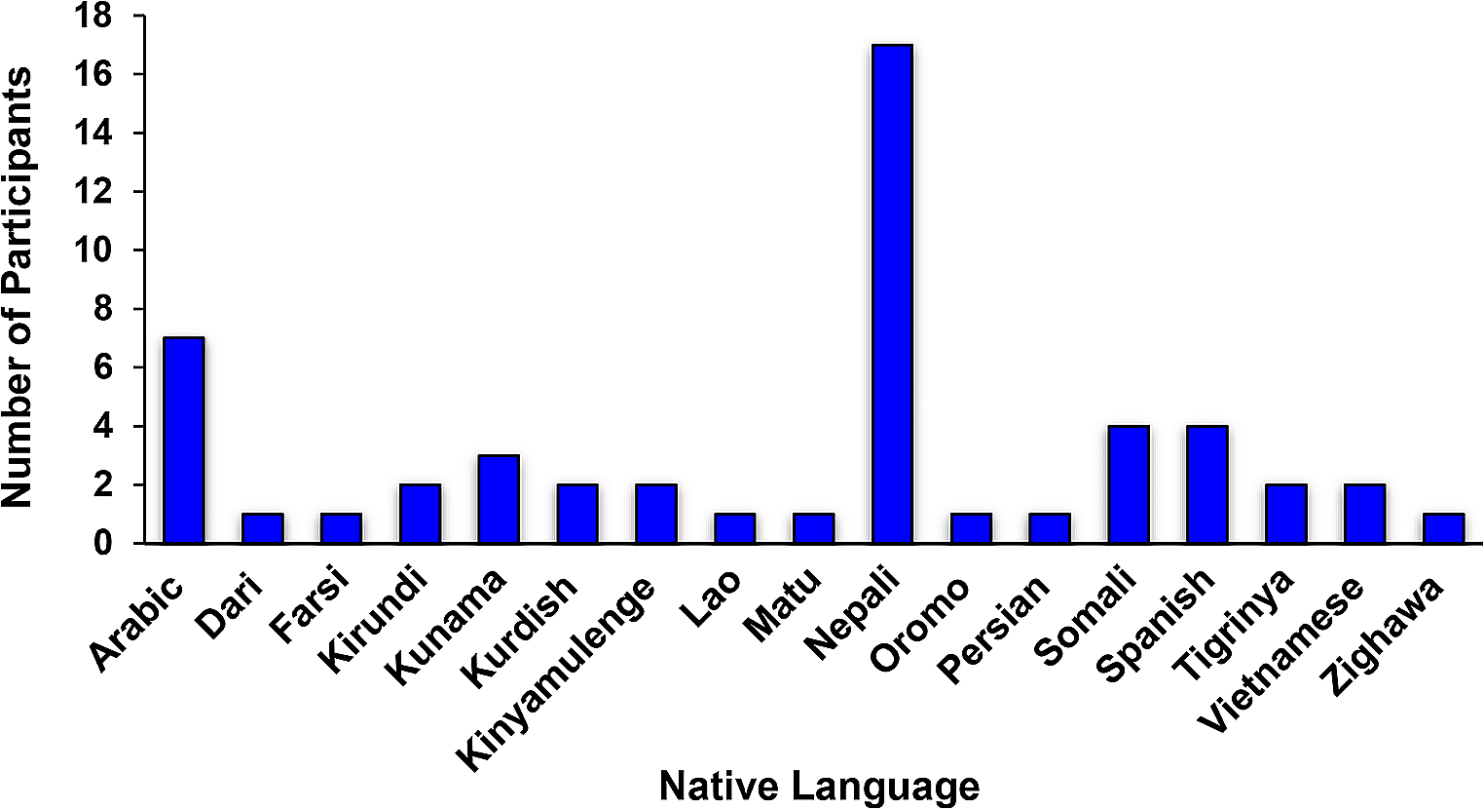
Native languages spoken by refugees (*N* = 52)

The highest group represented was Bhutanese (32.7%, 17/52), and the lowest (1.9%, 1/52), with equal representation were Colombian, Kenyan, Laotian, Mexican, Myanmar, Kenyan and Pakistani. Participants spoke 17 different languages with Nepali being the most common (Fig. 4).

The group consisted of 42.3% (22/52) men and 57.7% (30/52) women (Table 2). The mean (SD) age was 45.5 (15.5) years. The number of years of education was almost equally distributed among the 3 ranges: 0–5 years (34.6%, 18/52), 6–10 years (34.6%, 18/52), and 11–16 years (30.8%, 16/52). The majority of participants were covered under Medicaid (53.8%, 28/52), and 5.8% (3/52) had private dental insurance.

**Table 2:** Changes in participant (*N* = 52) responses before (pre-intervention) and 2 weeks after (follow-up) the oral health education program from the oral health practices and perceptions section of the sociodemographic survey and oral health perceptions of refugees

Oral Health Practices and Perceptions of Oral Health	Mean (SD)			
	Pre-Intervention	Follow-up		*P*-value
How many times do you brush per day?	2.24 (0.03)	2.79 (0.01)		0.01*
How many times do you clean between teeth per day?	1.79 (0.02)	277 (0.04)		0.00*
Are you happy with your teeth?	0.54 (0.05)	0.70 (0.03)		0.09
Have you been educated about oral health?	0.81 (0.03)	2.44 (0.01)		0.00*
Have you been treated by a dental hygienist?	0.65 (0.04)	0.69 (0.02)		0.82
Would you like to learn how to keep mouth and teeth healthy?	2.40 (0.01)	2.90 (0.05)		0.00*
How important is oral health?	2.12 (0.01)	2.90 (0.03)		0.00*
How important is oral health to your general health?	2.52 (0.07)	2.80 (0.04)		0.01*
Are you planning to visit a dental clinic in the next 6 months?	2.10 (0.09)	2.20 (0.09)		0.02*

Data are for participants who responded “Yes” to a question or selected the specific option

Numbers in parentheses represent the percentage of participants who answered “Yes” to the question or selected the specific option

*Some participants had more than one type of insurance

To investigate the demographics related to oral health and dental care access of refugees, data were obtained from the Sociodemographic Survey and Oral Health Perceptions of Refugees and presented by sections the questions were grouped into. Table 3 shows data from the first two sections of the survey that included Demographics and Dental History of participants.

**Table 3:** Summary of participant (*N* = 52) responses for the demographic and dental history sections of the sociodemographic survey and oral health perceptions of refugees survey

Survey Item	Number of Participants (%)
Demographic Characteristics	
1. Gender	
Male	22 (42.3)
Female	30 (57.7)
2. Age (years)	
19–30	11 (21.2)
31–50	21 (40.3)
51–76	20 (38.5)
3.Years of education	
0–5	18 (34.6)
6–10	18 (34.6)
11–16	16 (30.8)
4. Self-reported knowledge of English	
Poor	33 (63.5)
Fair	15 (28.8)
Good	4 (7.7)
5. Type of insurance*	
Dental	3 (5.8)
Medicaid	28 (53.8)
Private health	19 (36.5)
Medicare	10 (19.2)
**Dental history**	
Last dental visit	
Within current year	14 (26.9)
2–3 years	5 (9.6)
4–6 years	6 (11.5)
6–10 years	1 (1.9)
10 or more years	1 (1.9)
Never	25 (48.1)
Yearly dental checkup	7 (13.5)
Reasons for not seeking dental care	
Too expensive	25 (48.1)
No insurance	17 (32.7)
No access to dental care	3 (5.8)
Missing teeth	25 (48.1)
Mouth odor	15 (28.8)
Bleeding gums	22 (42.3)
Broken teeth	17 (32.7)
Tooth pain	22 (42.3)
Sensitive teeth	24 (46.2)
Loose teeth	13 (25.0)
Dry mouth	14 (26.9)

Data represents average score of responses to Likert-type questions

*Significant at *P* < 0.05

According to the results, only 13.5% (7/52) of participants had yearly dental checkups, and nearly half (48.1%, 25/52) had never been to the dentist. Most indicated they did not seek out preventive dental care because of lack of finances (55.6%, 25/52) or lack of dental insurance (37.8%, 17/52). Regarding current dental concerns, most indicated they had tooth pain (42.3%, 22/52), bleeding gums when brushing or flossing (42.3%, 22/52), broken teeth (32.7%, 17/52), missing teeth (48.1%, 25/52), sensitive teeth (46.2%, 24/52), or loose teeth (25.0%, 13/52). Over a quarter had dry mouth (26.9%, 14/52) or mouth odor (28.8%, 15/52).

### Oral Health Practices and Perceptions

The third section of the sociodemographic survey (Table 2) was related to self-reported Oral Hygiene Practices and Perceptions of Oral Health. The data from this section, which represents average score of responses to Likert-type questions, was analyzed to test our hypothesis that OHEP can improve oral health awareness and behaviors of refugees and to answer research questions about the effect of OHEP on oral health awareness and behaviors. Participants responded to surveys related to Oral Health Practices and Perceptions during Phase 1 (Pre-Intervention) and after the educational program (Follow-up). Two weeks after the OHEP, participants reported (Table 2) that they brushed more frequently (mean [SD] = 2.79 [0.01], *P* = 0.01), cleaned between teeth more frequently (2.77 [0.04], *P* < 0.01), felt that they were more educated about oral health (2.44 [0.01], *P* < 0.01), and were more interested in learning about keeping their mouth and teeth healthy (2.90 [0.05], *P* < 0.01). They also had a higher perception of the importance of oral health (mean [SD] = 2.90 [0.03], *P* < 0.01) and the importance of oral health to general health (2.80 [0.04], *P* = 0.01). More participants reported an intention to visit a dental clinic in the next 6 months (mean [SD] = 2.20 [0.09], *P* = 0.02).

### Word Assessment Test Using EOHL-BL40 Survey

**Fig. 5:**
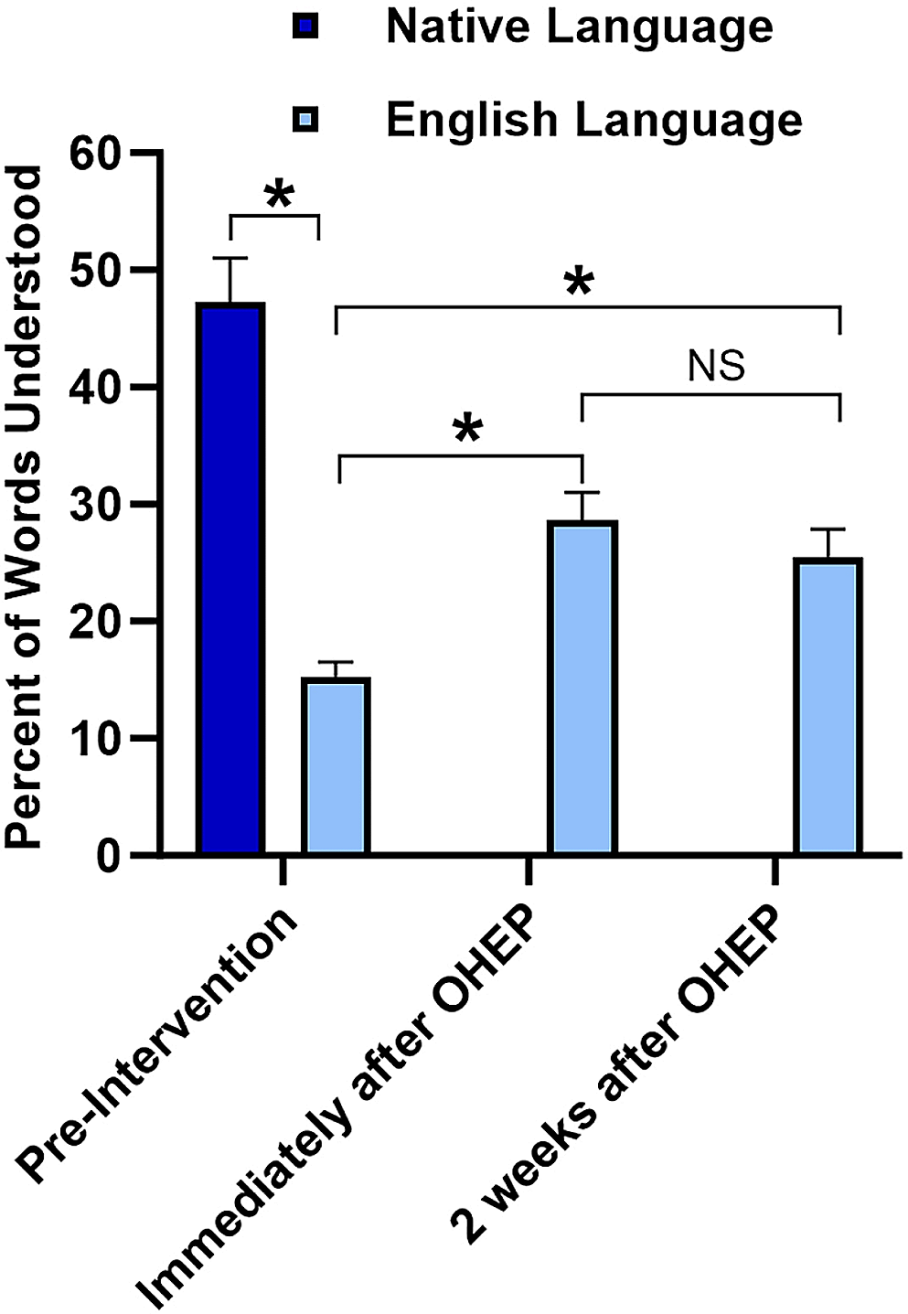
Percent of words understood in native language and in English at Pre-Intervention, and Immediately after or 2 weeks after Oral Health Educational Program (OHEP) in English. Data: Mean ± SE; N = 52; *P < 0.05; NS - not sgnificant difference

The 40-word recognition survey (EOHL-BL40) was used to evaluate the effects of the OHEP on the OHL of participants and their retention of information from the oral health educational program over time. We investigated whether the OHEP effected OHL of participants and whether there was a gap in OHL between English and native language. We hypothesized that OHEP will improve the OHL of participants. We also hypothesized that EOHL-BL50 pre-test in English and in native language will reveal a difference in the levels of OHL. Data are presented as percentage of known and understood words with SE (Fig. 5).

The percentage for words recognition (in both native and English language) before OHEP (Pre-intervention) was significantly higher in the participant’s native language (47.3 ± 3.70, *P* < 0.05) compared with survey in English (15.29 ± 1.24) Immediately after the OHEP, the Post-intervention score for words in English was significantly higher (28.61 ± 2.41, *P* < 0.05) compared with the Pre-intervention scores (15.29 ± 1.24). The Follow-up intervention score (2 weeks following OHEP) for words in English didn’t change much (25.48 ± 2.37) than Post-intervention but still it was significantly higher (*P* < 0.01) when compared with the scores at Pre-intervention (Fig. 5).

## Discussion

In the current study, we evaluated the effect of an OHEP on the OHL of immigrants. We hypothesized that (OHEP) can improve the OHL, oral health awareness and behaviors of refugees. We also anticipated that there will be difference between level of OHL between English and native languages. The results of the study were as expected. We knew from reviewing the literature that health literacy is low in the general population and especially the foreign born. What was surprising was the difference between the English OHL and OHL in native language. The results indicated that our OHEP had a positive effect on the perceptions of oral health and on personal oral hygiene practices of immigrants. The statistically significant changes in EOHL-BL40 scores after the OHEP also suggested participants had better OHL after the program. Since the OHEP was presented in English, the Post-intervention assessment of OHL was conducted in English only, and our results indicate participants had an increased comprehension of dentally related terms in English. Although scores for words understanding in English at Follow-up intervention showed a slight decrease in OHL, they were still significantly higher than Pre-intervention scores.

Low OHL has been identified as a major barrier to dental care [31]. Furthermore, it has been suggested that more dental professionals need to be involved in OHL research for development of OHEP to improve the OHL of patients [32–34]. Such programs are especially important for linguistically diverse individuals with limited knowledge of English who are at higher risk of low OHL than native English speakers [35]. It has been shown previously that immigrants with limited English proficiency achieve higher oral health literacy scores when tested in their native language [36] page 22 lines. In the current study, Follow-up intervention results on the oral health perceptions of refugees’ survey indicated our OHEP improved the awareness of oral health and personal oral hygiene practices of study participants, which suggested improvements in OHL. However, a study by Alrashdi et al. reported no such association with OHL at 3 to 6 months following administration of OHEP [37]. In contrast, in our study the OHEP and the evaluation of its effectiveness on knowledge and oral health perceptions was completed in 2 weeks. These differences in results suggest the periodic review of learned knowledge may be beneficial for long-term retention of information and maintenance of good oral hygiene habits. For individuals who have regular access to dental care, this reinforcement can occur during normal checkup every 6 months.

In the current study, the statistically significant Post-intervention and Follow-up intervention scores on the EOHL-BL40 suggested the OHEP increased our participants’ knowledge of terms related to oral health and dental care. However, unlike a similar study [14] that assessed OHL of participants, we evaluated word comprehension instead of word recognition which is a method previously incorporated by another study [23]. Some argue that word recognition assessments may be inadequate for determining a person’s true OHL level [22–24]. . Our results support word comprehension instruments for assessment of OHL.

We hypothesized that there will be a difference between OHL in English and native language in the Pre-intervention phase. To test the hypothesis, we used translated versions of the EOHL-BL40 in the participant’s native language to add an additional assessment level of word comprehension for bilingual individuals. Our results indicated there was a gap in OHL between comprehension of dental terms in English and the participant’s native language. This finding corresponded with results of a similar study, where participant had significantly higher health literacy levels when immersed in communication using native language when compared to English [36].

### New Contribution to the Literature

Our study adds to the scarce body of literature on the topic of OHEP impact on OHL and oral health perceptions and practices of refugees. The research was unique as it included assessment of OHL of participants in their native language as well as in English, which resulted in significant finding of an OHL gap between two different languages. For professionals who are developing education programs and OHL assessments for foreign born who are bilingual in English, it is important to keep in mind that OHL may be higher if evaluated in the native language. This finding may warrant additional research into methods that can close the gap. Moreover, the results of the current study support previous research that incorporated comprehension with OHL assessment of individuals, and show improvement in oral health perceptions and oral health practices of participants after the OHEP.

For future OHEP development for particular ethnic groups, perhaps incorporating equivalent foreign language terms into the OHEP presentation would improve the learning experience and increase the dental term vocabulary of participants. Given the differences in word comprehension between languages, future development of OHEP in English language could incorporate strategies that pre-assess participant understanding of planned program content. Evaluation of existing OHL could be delivered in multiple languages and may require collaboration with interpreters and community organizations, such as the IISTL in the current study, to ensure linguistically appropriate content delivery. Other studies have shown that interprofessional collaboration between healthcare professionals and institutions that assist with integration of immigrants into the community is effective and beneficial [38, 39].

The current study had several limitations. Because we used convenience sampling to recruit participants, our results may be affected by selection bias, which limits the generalizability of findings. However, our recruitment strategy was necessary given the availability of refugees who met study criteria. A related limitation was our small sample size. Our original recruitment goal was 98 participants. However, it was difficult to retain participants throughout the study, which explains our sample size of 52.

Although participants were proficient in English at an intermediate level, as determined by results of standardized test administered previously by IISTL some self-reported lower level of proficiency. This phenomenon was investigated in a study by Edele and colleagues where findings revealed that subjective estimates of language skills can be inadequate in comparison to objective test scores [40]. While most individuals completed the surveys independently, some required assistance with navigation of the surveys by clarification of instructions. This additional assistance was most needed for completion of the sociodemographic survey, despite its bilingual format. In addition to the challenges of survey administration, our word comprehension results for the EOHL-BL40 may have been limited by the self-report nature of participant responses. However, a pre-study calibration delivered prior to the session focused on explaining the meaning of comprehension by giving an example of the word “sugar” and the knowledge that it is sweet, not just recognizing the word. The difference between comprehension and word recognition was further explained by giving multiple examples and engaging the audience.

## Conclusion

Results of the current study suggest that a culturally appropriate OHEP presented in English may positively affect the OHL of refugees and immigrants, improve their oral health awareness, personal oral hygiene practices, and perceptions about the importance of oral health. The results for the EOHL-BL40 in English and in the native language of participants identified a gap in knowledge of dental terms between the 2 languages. This finding may suggest that, although the participants did not understand the meaning of a term in English, they understood the meaning when the term was presented in their language. As such, this identified gap in knowledge may be less about understanding the meaning of a term in one’s own language and more about not knowing the equivalent word in English. Because OHL is important for overall health, particularly in vulnerable populations such as immigrants and refugees, development of OHEP may require extensive research into the sociodemographic and historical oral health needs, beliefs, and practices of ethnically diverse groups to adjust the program for maximum effectiveness. As discussed by Nakazono and colleagues, measures related to sociodemographic predictors of perceived benefits of preventive practices and seriousness of oral health disease is a factor among ethnically diverse individuals [41]. In addition, interprofessional collaborations with local community leaders and organizations serving immigrant groups may improve understanding of ethnic populations and facilitate delivery of culturally appropriate educational programs by oral health professionals.

### Electronic supplementary material

Below is the link to the electronic supplementary material.

Supplementary material 1
