# Supplementary material for: Impact of an Oral Health Education Program on the Oral Health Literacy of Refugees
Source: J Immigr Minor Health. 2024 Apr 8;26(4):699–710. doi: 10.1007/s10903-024-01594-6 (PMC11289270; doi:10.1007/s10903-024-01594-6)
Supplement: Supplementary file 1 — Supplementary material 1 [file 10903_2024_1594_MOESM1_ESM.docx]

**Supplemental Table 1.** Sociodemographic Survey and Oral Health Perceptions of Refugees Survey Presented to Participants.

| **Survey Item** | **Response Type** | **Response Categories** |
| --- | --- | --- |
| **Demographic Characteristics** |  |  |
| 1. Gender | Dichotomous | female or male |
| 1. Age | Write in |  |
| 1. Time residing in United States | Write in |  |
| 1. Nationality | Write in |  |
| 1. Years of education | Write in |  |
| 1. Self-reported knowledge of English | Multiple choice | good, fair, or poor |
| 1. Dental insurance holder | Dichotomous | yes or no |
| 1. Medicaid recipient | Dichotomous | yes or no |
| 1. Health insurance holder | Dichotomous | yes or no |
| 1. Medicare recipient | Dichotomous | yes or no |
| **Dental History** | |  |
| 1. Last dental checkup | Multiple choice | in 2015, 2-3 years ago, 4 – 6 years ago, 6-10 years ago, 10+ years ago, or never |
| 1. Yearly dental checkup | Dichotomous | yes or no |
| 1. Reasons for not seeking dental care | Multiple choice | too expensive, no insurance, fear, no access, or it is not important |
| 1. Missing teeth | Dichotomous | yes or no |
| 1. Mouth odor | Dichotomous | yes or no |
| 1. Bleeding gums | Dichotomous | yes or no |
| 1. Broken teeth | Dichotomous | yes or no |
| 1. Tooth pain | Dichotomous | yes or no |
| 1. Sensitive teeth | Dichotomous | yes or no |
| 1. Loose teeth | Dichotomous | yes or no |
| 1. Dry mouth | Dichotomous | yes or no |
| **Oral Health Practices and Perceptions of Oral Health** | |  |
| 1. How many times do you brush your teeth per day? | Likert-like | 2x day, 1x day, sometimes, or never |
| 1. How often do you clean between your teeth? | Likert-like | 2x day, 1x day, sometimes, or never |
| 1. Are you happy with your teeth? | Dichotomous | yes or no |
| 1. Have you been educated about oral health? | Likert-like | yes, maybe, no, or I do not know |
| 1. Have you been treated by a dental hygienist? | Likert-like | yes, maybe, no, or I do not know |
| 1. Would you like to learn how to keep your mouth and teeth healthy? | Likert-like | yes, maybe, no, or I do not know |
| 1. How important is oral health? | Likert-like | very important, important, somewhat important, or not important |
| 1. How important is oral health to your general health? | Likert-like | very important, important, somewhat important, or not important |
| 1. Are you planning to visit a dental clinic within the next 6 months? | Likert-like | yes, maybe, no, or I do not know |
